# The *Prrx1* limb enhancer marks an adult subpopulation of injury-responsive dermal fibroblasts

**DOI:** 10.1242/bio.043711

**Published:** 2019-07-05

**Authors:** Joshua D. Currie, Lidia Grosser, Prayag Murawala, Maritta Schuez, Martin Michel, Elly M. Tanaka, Tatiana Sandoval-Guzmán

**Affiliations:** 1 CRTD-Center for Regenerative Therapies Dresden, Technische Universität Dresden, Fetscherstrasse 105, 01307 Dresden, Germany; 2 Department of Cell and Systems Biology, University of Toronto, 25 Harbord Street, M5S 3G5 Toronto, Canada; 3 Research Institute for Molecular Pathology (IMP), Vienna Biocenter (VBC), Campus-Vienna-Biocenter 1, 1030 Vienna, Austria

**Keywords:** Lineage tracing, Dermal fibroblast, Wound healing, Limb progenitor

## Abstract

The heterogeneous properties of dermal cell populations have been posited to contribute toward fibrotic, imperfect wound healing in mammals. Here we characterize an adult population of dermal fibroblasts that maintain an active *Prrx1* enhancer which originally marked mesenchymal limb progenitors. In contrast to their abundance in limb development, postnatal *Prrx1* enhancer-positive cells (Prrx1^enh+^) make up a small subset of adult dermal cells (∼0.2%) and reside mainly within dermal perivascular and hair follicle niches. Lineage tracing of adult Prrx1^enh+^ cells shows that they remain in their niches and in small numbers over a long period of time. Upon injury however, Prrx1^enh+^ cells readily migrate into the wound bed and amplify, on average, 16-fold beyond their uninjured numbers. Additionally, following wounding dermal Prrx1^enh+^ cells are found out of their dermal niches and contribute to subcutaneous tissue. Postnatal Prrx1^enh+^ cells are uniquely injury-responsive despite being a meager minority in the adult skin.

## Abstract (precis)

**Summary:** Dermal Prrx1-enhancer cells are injury-responsive fibroblasts. Prrx1enh+ cells readily migrate into the wound bed and amplify on average 16-fold beyond their uninjured numbers.

## INTRODUCTION

The skin is the largest organ and one with a crucial task: making a multifunctional barrier between internal organs and the outside environment. The regenerative capacity of the skin is essential to maintain its integrity. However in adult skin, wound healing typically results in scar tissue. In the search for therapies that enhance wound healing or reduce fibrosis, cellular heterogeneity has emerged as an added layer of complexity that profoundly shapes the outcome of wound responses (Driskell et al., 2013b). Fibroblast heterogeneity is particularly important since fibroblasts predominantly determine whether unresolved fibrotic scars form during wound healing (Gurtner et al., 2008). Central to this idea is that mixed populations of cells may carry intrinsic differences in their response to wound-related signals or their capacity to reconstitute all the structures of the intact organ.

Several groups have identified fibroblast subpopulations by a single or battery of molecular markers. Cells derived from Engrailed-1 embryonic lineage (Rinkevich et al., 2015) or adult cells with *Gli-1*^+^ expression have been shown to contribute to wound fibrosis (Kramann et al., 2014), while Wnt-dependent expansion of BLIMP1^+^ dermal cells can support *de novo* hair follicle formation during wound repair (Kretzschmar et al., 2014). Ideally, such categorization would distinguish subpopulations with higher regenerative or differentiation potential that could be analyzed in isolation from fibrosis-associated cells. The final goal would be to amplify and recruit non-fibrotic populations during wound repair, or inversely, deter fibrotic cells from making contributions to wound healing.

To identify adult cells that retain a progenitor-like ability to participate in tissue formation, we looked at molecular markers that are present during organogenesis. One such marker is the transcription factor paired-related homeobox 1 (*Prrx1* or *Prx1*), an early marker of lateral plate mesoderm (LPM) that labels progenitors of nascent limb skeleton and soft connective tissue of the flank and limb (leussink et al., 1995). *Prrx1* loss-of-function mutants do not survive after birth and show severe defects in the formation of skull, limb and vertebrae (Martin et al., 1995). Additionally, *Prrx1* is upregulated following salamander limb amputation (Satoh et al., 2007) as well as in anuran limb regeneration (Suzuki et al., 2005). Transgenic mouse models of *Prrx1* expression rely on a specific enhancer that encompasses approximately 2.4 kb upstream of the transcriptional start site (Logan et al., 2002; Martin and Olson, 2000). In reporter lines, this enhancer was used to drive LacZ or Cre recombinase expression in embryonic lateral soft connective tissue, portions of craniofacial mesenchyme, and limb skeleton and connective tissue. A recent report implicated a population of PRRX1^+^ cells in the regeneration of calvarial bone (Wilk et al., 2017), but whether PRRX1 protein (PRRX1^+^) or enhancer activity (Prrx1^enh+^) remain postnatally in other tissues is unknown. This led us to investigate PRRX1 protein expression and enhancer activity in the skin to determine its role in homeostasis and tissue repair.

## RESULTS

### PRRX1 protein marks a broad population of limb-bud progenitors and adult mesenchymal dermal cells

**Fig. 1.**
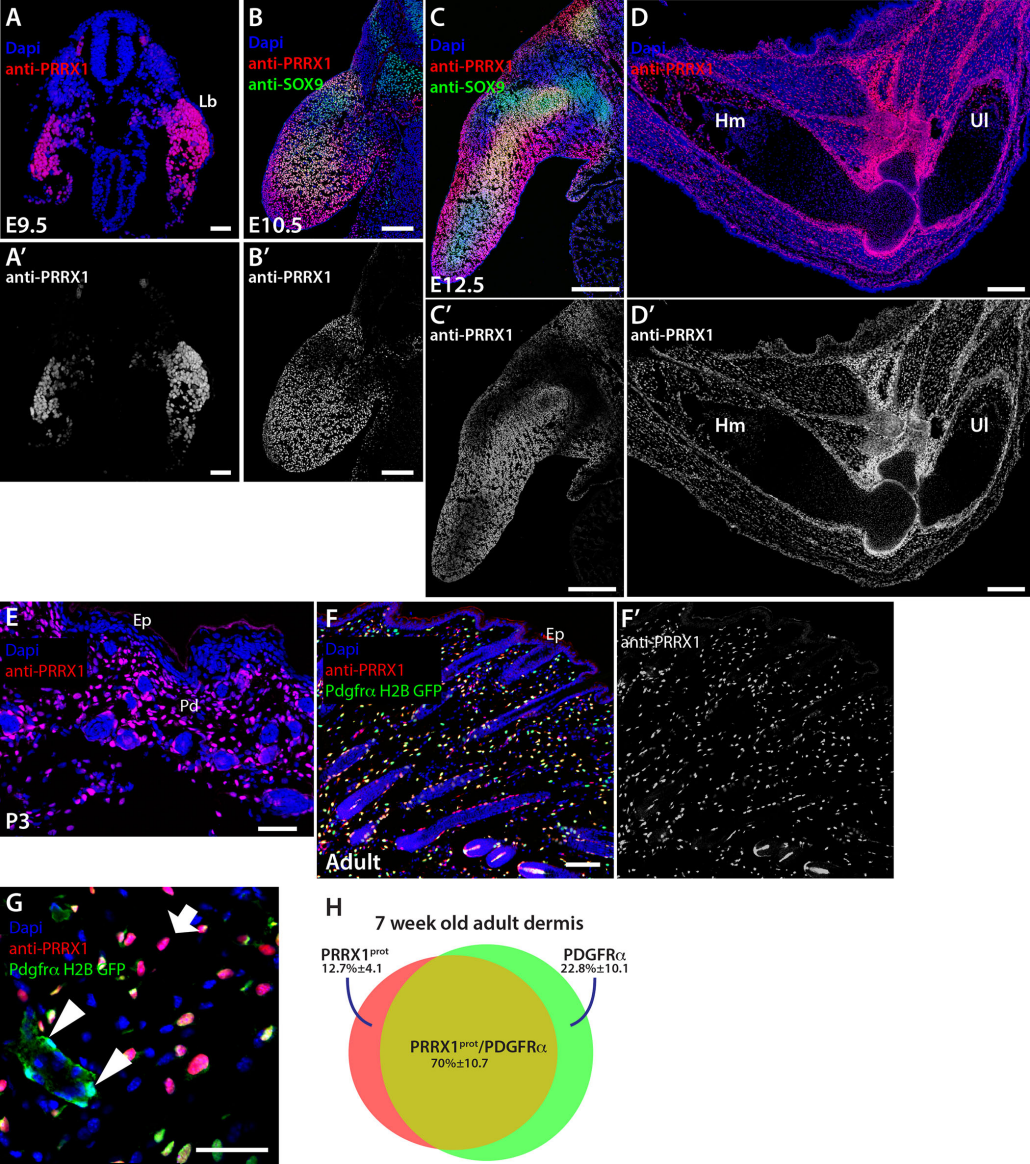
**PRRX1 protein marks a broad mesenchymal population during limb development and in adult dermal tissue.** (A,A′) Representative micrographs of antibody staining against PRRX1 protein. The peak of PRRX1 in the limb bud (Lb) is around E9–10. Nuclei in blue, PRRX1 antibody staining in red, greyscale in A′. Scale bars: 50 μm. (B,B′) At E10.5, cartilage condensations positive for SOX9 protein (in green), in the midline of the limb downregulate PRRX1 protein. Scale bars: 200 μm. (C,C′) By E12.5, skeletal condensations are distributed along the limb and downregulate PRRX1. Scale bars: 500 μm. (D,D′) At E16.5, the limb has patterned the musculo-skeletal elements, humerus (Hm), the clear elbow joint, ulna (Ul) and digits. PRRX1 is highest at the elbow area. Scale bar: 200 μm. (E) After birth, at P3, PRRX1^+^ cells are still present across dermis, including reticular and papillary dermis (Pd). Epidermis (Ep) is negative for PRRX1. Scale bar: 50 μm. (F) In adult skin, PRRX1^+^ cells in red, (greyscale in F′) are compared to the population of PDGFRα^+^ cells in green and quantified (H). Scale bar: (F) 200 μm. (G) High magnification of adult skin. Arrow marks PDGFRα^+^ cells that are PRRX1^−^. Arrowheads mark PDGFRα^−^ cells that are PRRX1^+^. Scale bar: 50 μm. (H) Quantification of the PDGFRα and PRRX1 populations in adult dermis, represented in a Venn diagram. The mean percentage of cells/mm^2^±s.d. is reported.

*Prrx1* was originally characterized as a progenitor marker of limb skeleton and soft connective tissue using a combination of *in situ* hybridization and Cre activity or LacZ expression in reporter mice (Durland et al., 2008; Martin and Olson, 2000). However, a precise timeline of protein expression at both embryonic and postnatal timepoints is unknown. To do this, we used a previously characterized polyclonal antibody anti-PRRX1 (Gerber et al., 2018; Oliveira et al., 2017). By immunohistochemistry, PRRX1**^+^** cells were detected in limb bud and lateral plate at embryonic day (E) 9.5, where most mesenchymal cells are positive (Fig. 1A,A′). At this stage, PRRX1 protein can be found throughout the mesenchyme at what is considered the beginning of the budding phase. At E10.5 the limb bud is defined and protruding from the body flank (Fig. 1B,B′). At E12.5, cartilage condensations become evident, with cells within the condensate (SOX9+ cells) downregulating *Prrx1* expression. However, most mesenchymal cells still remain PRRX1**^+^** (Fig. 1C,C′).

At E16.5, clear PRRX1**^+^** and PRRX1**^−^** zones were visible in the limb, although most connective tissue cells were still PRRX1**^+^** (Fig. 1D,D′). We further investigated if PRRX1 remains in postnatal tissue or if its expression is restricted to embryonic and neonatal stages. In postnatal day (P) 3, PRRX1**^+^** cells persist abundantly in the dermis (Fig. 1E). Since PDGFRα has been previously suggested as a pan marker of dermal fibroblasts (Driskell et al., 2013b), we used the *Pdgfrα-H2B-EGFP* transgenic mouse to quantify the overlap of PRRX1**^+^** in adult mesenchymal dermal tissue (Fig. 1F,F′). We found that in 7 week-old mice 12.7%±4.1 (*n*=6) of total PRRX1**^+^** cells are PDGFRα**^−^**. Conversely, 22.8%±10.1 of the total PDGFRα**^+^** cells are PRRX1**^−^** (Fig. 1G,H). This result was consistent with data from Rinkevich et al., who found that a considerable percentage of *Engrailed-1* lineage fibroblasts do not express *Pdgfrα* (Rinkevich et al., 2015). Neither PRRX1 nor PDGFRα encompassed the full complement of mesenchymal cells and *Pdgfrα* is also expressed in non-mesenchymal lineages such as megakaryocytes and platelets (Demoulin and Montano-Almendras, 2012; Ye et al., 2010). Therefore, our data suggest that PRRX1 is an optimal marker to demarcate a broad mesenchymal population in the dermis.

### The *Prrx1* enhancer labels embryonic mesenchymal progenitor cells and a small subset of the broad adult PRRX1^+^ dermal cell population

**Fig. 2.**
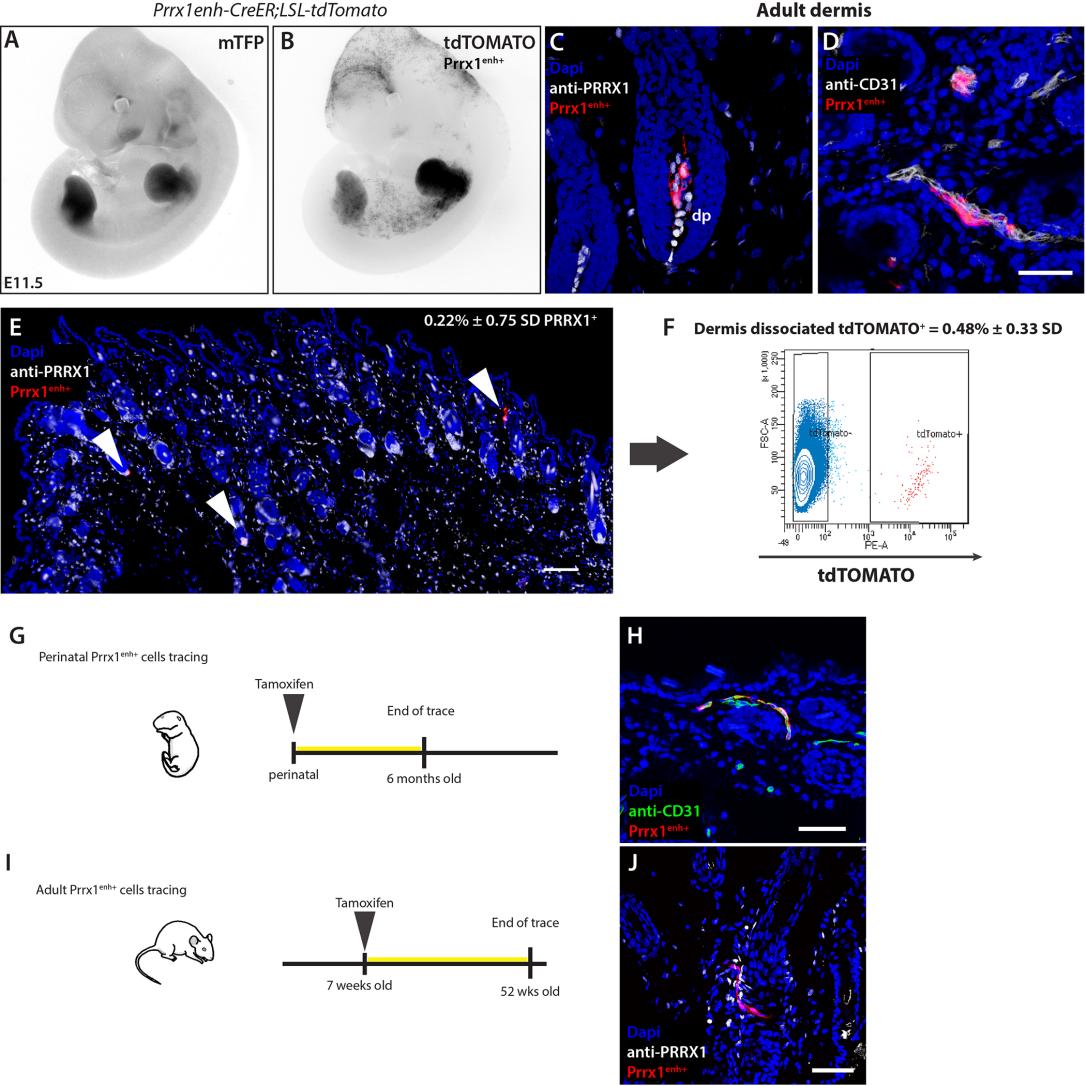
**The *Prrx1* enhancer is active in limb-bud mesenchyme progenitors and adult dermis.** To lineage trace the fate of Prrx1^enh+^ cells, a transgenic *Prrx1 enhancer-CreER-T2A-mTFPnls* was crossed to a reporter line *Rosa-CAG-loxP-stop-loxP-tdTomato* (referred to as *Prrx1enh-CreER;LSL-tdTomato*). (A) Inverted greyscale images of embryos showing limb TFP expression at E11.5 under the *Prrx1* enhancer. (B) Upon tamoxifen administration (1 mg oral gavage to mothers 24 h prior to imaging), tdTOMATO^+^ (Prrx1^enh+^) is visible in limb buds. Additionally, Prrx1^enh+^ cells are present in a salt-and-pepper pattern in inter-limb flank, as well as cranial and craniofacial mesenchyme. (C,D) Prrx1^enh+^ cells in adult limb dermis. Cre recombinase activity labels cells primarily in dermal papilla (dp) and perivascular cells. Scale bar: 50 μm. (E) An overview of the sparsely-labeled cells within the adult limb dermis. Arrowheads mark Prrx1^enh+^ positive cells that make up 0.22%±0.75 s.d. of PRRX^+^ cells. Scale bar: 200 μm. (F) Dermal dissociation of cells 3 weeks after tamoxifen administration and FAC sorting of tdTOMATO^+^ cells shows that in adult skin only a small population of cells retain the activity of the *Prrx1* enhancer. (G) Graphic representation of experimental setup to analyze the role of perinatal Prrx1^enh+^ cells in tissue homeostasis. (H) A representative tissue section of experiment (G) after 6 months. Scale bar: 50 μm. (I) Graphic representation of experimental setup to analyze the role of postnatal Prrx1^enh+^ in tissue homeostasis. (J) A representative micrograph of experiment (I) after 1 year. Scale bar: 50 μm.

To trace the fate of PRRX1 cells in homeostasis and injury, we generated transgenic mice expressing Cre-ERT under the control of the 2.4 kb *Prrx1* enhancer (Logan et al., 2002) together with nuclear teal fluorescent protein (TFP) as a reporter. We next crossed the *Prrx1 enhancer-CreER-T2A-mTFPnls* mice with *Rosa-CAG-loxP-stop-loxP-tdTomato* mice (referred to as *Prrx1enh-CreER;LSL-tdTomato*) (Fig. 2A), allowing us to trace the fate of *Prrx1* enhancer-positive cells (Prrx1^enh+^) upon tamoxifen administration. A single low dose of tamoxifen (1 mg, gavage) to gestating mothers 24 h prior to collection at E10.5 yielded labeling of Prrx1^enh+^ cells in a majority of limb bud cells, facial mesenchyme and inter-limb flank after 24 h (Fig. 2B). In postnatal and juvenile mice, the conversion of the *LSL-tdTomato* reporter by *Prrx1* enhancer-driven CreER was vastly reduced such that even after repeated doses of tamoxifen (5 mg, gavage), only a small subset of cells was visible within the dermis and scattered in other connective tissues of the limb (Fig. 2E). Labeled Prrx1^enh+^ cells were found primarily within two specialized dermal niches: the hair follicle dermal papillae (dp) at 84% (Fig. 2C) and in the perivascular space (Fig. 2D). Prrx1^enh+^ cells were also occasionally found in the dermal sheath of hair follicles and in papillary dermis. We confirmed in tissue sections that all Prrx1^enh+^ cells in the adult dermis were also PRRX1^+^. Prrx1^enh+^ cells made up just 0.22%±0.75 s.d. (*n*=6) of the total PRRX1^+^ mesenchymal cells in the adult limb dermis. Likewise, dissociation of the dermis and FAC sorting revealed that approximately 0.48%±0.33 s.d. of dermal cells were tdTOMATO^+^ (Fig. 2F). We also confirmed that the higher conversion dose required in dermis of young adult mice was not due to the oral administration, since a single dose of tamoxifen was sufficient to observe high conversion in other areas where the enhancer is active: ears, mammary glands and the intermaxillary segment of the upper lip (Fig. S1A–C). Furthermore, we dissociated dermal cells from *Prrx1enh-CreER;LSL-tdTomato* mice, to allow *in vitro* conversion of Prrx1^enh+^ cells and found less than 1% of converted cells (data not shown). Thus, the Prrx1^enh+^ broadly labels embryonic limb-bud mesenchyme which reaches its peak before patterning of differentiated limb tissues, but the number of cells that preserve the activity of the enhancer rapidly declines after birth. In contrast to the embryonic congruity of enhancer and protein, in adulthood, the Prrx1^enh+^ population becomes a small subset of the broad PRRX1 population.

An open question was if cells with active *Prrx1* enhancer at late embryonic and early postnatal periods would have a role in building and maintaining adult tissue. We administered tamoxifen perinatally and examined if Prrx1^enh+^ lineage cell number would increase over time and in a clonal manner in specific dermal niches (Fig. 2G). We found that although the number of cells converted perinatally is higher than in adult, no clonal expansion was observed after 6 months (Fig. 2H). The same result was obtained when recombination was induced in 7-week-old mice and tissue was collected 1 year later (Fig. 2I,J). We next investigated if Prrx1^enh+^ adult cells might play a role in injury repair.

### *Prrx1* enhancer cells are injury-responsive and amplify upon limb full-thickness skin wounding

We were curious if these limited Prrx1^enh+^ cells in the adult mouse retained embryonic-like properties that could contribute positively to repair and regeneration. Previously, the mouse *Prrx1* enhancer was shown to be active during wound healing and spike formation in *Xenopus laevis* (Suzuki et al., 2007), but absent in wound healing of mouse back skin (Yokoyama et al., 2011), the predominant experimental paradigm for skin healing in the mouse. Although PRRX1 protein is present in dermal cells of back skin, we could detect no more than one or two Prrx1^enh+^ cells in multiple sections of at least 4 mm expanding along the back (Fig. S2A) (*n*=10 animals). The lack of enhancer-positive cells in back skin was confirmed after tamoxifen administration in embryos, adult mice, or after injury.

**Fig. 3.**
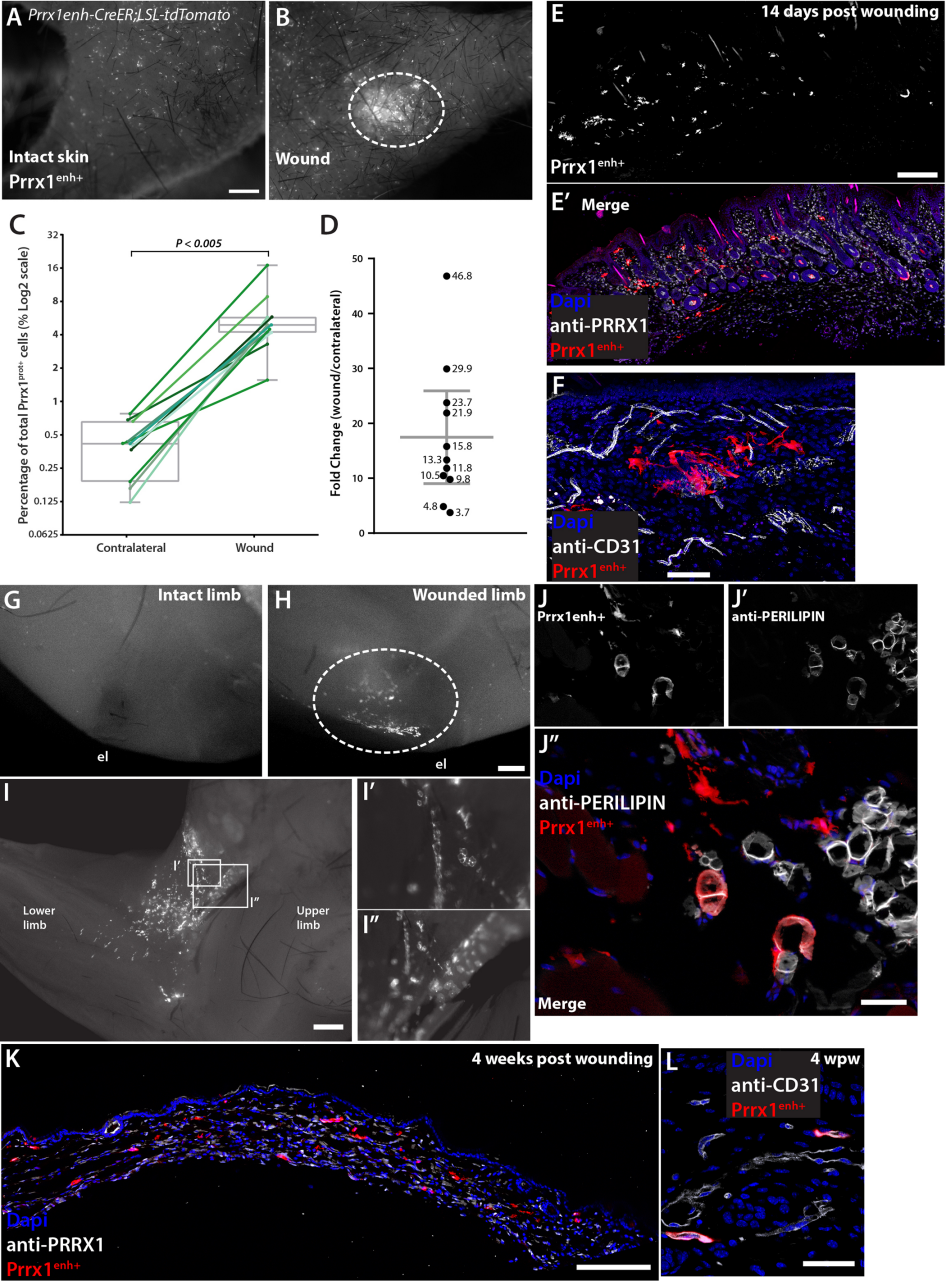
**Prrx1^enh+^ cells amplify after injury in adult skin.** (A) Stereoscope micrograph of skin from an intact limb. (B) Skin from wounded limb 21 days after wounding. Prrx1^enh+^ cells respond to 2 mm full-thickness wounds in the limb, highlighted by the dashed oval. Scale bar: 1 mm. (C) Quantification of Prrx1^enh+^ cells in sections from paired samples of contralateral and wounded limb skin. The percentage of Prrx1^enh+^ from the total population of PRRX1^+^ cells/mm^2^ (*n*=11, box is mean and 95% CI, error bars min and max values). Y-axis is shown in log2 for optimal visualization of values below 1. Paired *t*-test, two tailed: *P*<0.005. (D) Fold change of Prrx1^enh+^ cells/mm^2^ over their paired contralateral limb. Bar value is mean with error bars representing 95% CI. (E,E′) A representative skin section of a wound 21 days after wounding. Scale bar: 200 μm. (F) Prrx1^enh+^ cells in the wound bed. Cells are not associated to blood vessels during the wound-resolving phase. Scale bar: 100 μm. (G) Fixed limb of mouse after skin collection in contralateral limb (el) elbow. (H) Fixed limb of mouse after skin collection in wounded limb. Dashed oval marks the tissue under the wounded skin. Scale bar: 500 μm. (I–I″) Wounded limb with inserts showing detail of Prrx1^enh+^. Scale bar: 1 mm. (J–J″) Representative section of the limb subcutaneous tissue under the wound. In white is the adipocyte marker PERILIPIN. Scale bar: 50 μm. (K) At 4 weeks post wounding, once most inflammation has resolved, Prrx1^enh+^ cells remain in the wound bed. Scale bar: 200 μm. (L) At 4 weeks post wounding Prrx1^enh+^ return to an association with blood vessels. Scale bar: 50 μm.

Given that the *Prrx1* enhancer marks limb progenitors, and fibroblasts are known to carry unique positional information based on their location in different body parts (Chang et al., 2002), we decided to lineage trace Prrx1^enh+^ cells in a limb full-thickness skin injury model. We performed 2 mm full-thickness skin wounds in the upper limbs of *Prrx1enh-CreER;LSL-tdTomato* (Fig. S2B). Mice were wounded 3 weeks after the last administration of tamoxifen to prevent recombination during wound healing. In contrast to back skin wounds, it was not possible to splint limb skin wounds. A semi-occlusive bandage (Tegaderm 3M) was applied for the first 48–60 h to prevent infection. This bandage only mildly impedes wound contraction that is characteristic of skin wounds in rodents (Fig. S2C). Fourteen days post wounding (14 dpw), animals were euthanized and the wounded and contralateral limb were compared for the percentage of Prrx1^enh+^ (tdTOMATO^+^) cells. At low magnification there was already an obvious amplification of Prrx1^enh+^ cells and their descendants within the wound bed compared with the adjacent wound margin and contralateral limb (Fig. 3A,B). Within 1 mm^2^ cross-sections of the wound (Fig. 3E,E′), despite an increased density of mesenchymal PRRX1^+^ cells by 1.3-fold, the percentage of labeled Prrx1^enh+^ lineage cells were on average 16.5-fold increased (±12.58 s.d., *P*=0.0015) over the uninjured contralateral dermis (Fig. 3C,D). This represents an increase of Prrx1^enh+^ from 0.37%±0.2 s.d. to 5.76%±3.8 s.d. of the total PRRX1^+^ cells. During the injury response, Prrx1^enh+^-derived cells were not necessarily associated to blood vessels (Fig. 3F), but primarily within the upper or papillary dermis. These results were confirmed in three independent experiments.

An important question to address was if other subpopulations of dermal cells would also amplify in response to injury in a similar manner as Prrx1^enh+^ cells. To address this, we used a transgenic mouse expressing inducible CreER under the control of a 17-kb fragment of the *Col1a2* upstream enhancer crossed with a *Rosa-CAG-loxP-stop-loxP-tdTomato* mice (referred to as *Col1a2enh-CreER;LSL-tdTomato*). This transgenic mouse has been used previously as a reporter of wound healing in dorsal wounds (Higashiyama et al., 2011; Rajkumar et al., 2006). We administered tamoxifen 3 weeks before wounding and quantified tdTOMATO^+^ cells in contralateral and wound tissue (Fig. S3A,B). In uninjured tissue, Col1a2^enh+^ cells labeled dermal fibroblasts as well as portions of surface and interfollicular epithelia (Fig. S3A,C). No difference was found in the percentage of Col1a2^enh+^ cells among the total of mesenchymal PRRX1^+^ cells in injured and intact skin (Fig. S2D). In contrast to Prrx1^enh+^ cells, we found no enrichment of positive dermal cells within the wound bed or in the subcutaneous space under the wounds (Fig. S2E–H). In fact, there was a small depletion of Col1a2^enh+^/PRRX^+^ within the wound 21 days after injury. This suggests that the dermal cells labeled by Col1a2^enh+^ do not expand in response to injury, and rather are displaced by the majority of other dermal subpopulations, including Prrx1^enh+^ cells. These results were confirmed in three independent experiments.

### In response to injury*,* dermal *Prrx1*-enhancer cells contribute to tissues beyond dermal compartments

When collecting skin for tissue processing, we observed a concentration of Prrx1^enh+^ lineage cells in subcutaneous connective tissue surrounding the muscle immediately under the wounded skin (Fig. 3G–I″). We found that Prrx1^enh+^-derived cells were located within fascia, loose connective tissue and adipose tissue. We confirmed the contribution to adipose tissue by co-staining Prrx1^enh+^ with a PERILIPIN antibody (Fig. 3J–J″). In our experimental layout, tamoxifen is administered 5 weeks prior to tissue collection and recombination labels only a dispersed, small subset from the broad PRRX1^+^ population in the unwounded or contralateral limb. This suggests that upon injury, labeled, dermal-associated cells migrate from the dermis and take up residence in subcutaneous tissue where they contribute to diverse tissue types such as adipose tissue. After 4 weeks, when the 2 mm wound is resolved and cellular content is decreased, Prrx1^enh+^ cells remain in the wound bed (Fig. 3K) where they re-acquire an association with blood vessels (Fig. 3L). The number of cells in the wound derived from Prrx1^enh+^ cells after 4 weeks remains elevated compared to contralateral uninjured skin by a 11.48-fold increase (±4.1 s.d., *n*=6). The amplified number of Prrx1^enh+^ progeny in the wound suggests that cells that have an active enhancer under homeostatic conditions readily migrate into the wound bed and proliferate relative to other PRRX1^+^ cells. The activity of the *Prrx1* enhancer during wound healing was also assessed by administering tamoxifen 5 days after wounding. We found that in this context a single dose of tamoxifen was sufficient to detect Prrx1^enh+^ cells in the wound bed, suggesting that during the injury response the enhancer could be more active or previously negative cells could activate the enhancer. When quantifying the number of Prrx1^enh+^ cells in the wound bed at 14 days post wounding and 9 days post conversion, we found 4.8%±2.1 s.d. (*n*=5) of positive cells. Because one dose of tamoxifen fails to convert cells in uninjured skin, we were unable to calculate a fold-change between wound and contralateral conditions. However, the percentage of positive cells converted during healing is comparable to the percentage of positive cells in the wound bed from our pre-conversion experiments (5.76%±3.8 s.d.). This shows that the *Prrx1* enhancer remains active during wound healing, as evidenced by the ease of tamoxifen conversion during the early proliferative phase of wound repair.

## DISCUSSION

Mesenchymal cells are the primary drivers of fibrosis and scar formation after injury. Recent evidence suggests that rather than being a homogenous population of cells, dermal mesenchyme populations arise from diverse embryonic origins, occupy specific homeostatic niches and can be identified by their unique molecular profiles (Driskell et al., 2013a; Longaker and Chan, 2017; Rinkevich et al., 2015). It has been hypothesized that heterogenous cell populations may have intrinsic properties that favor either fibrosis or complete regeneration (Rinkevich et al., 2015). One illustrating example is the ability of digit tip fibroblasts in the P3 phalanx to regenerate versus non-regenerative fibroblasts in the P2 phalanx (Wu et al., 2013). Thus, identifying different sub-populations of mesenchymal cells is important to understanding how diverse cells influence injury repair.

We hypothesized that molecular attributes of embryonic mesenchyme might label adult cells with an enhanced propensity for self-renewal, repair and regeneration. We focused on *Prrx1* (Prx1), a homeobox-containing gene that marks mesenchymal progenitors of the limb, flank and craniofacial regions. *Prrx1* upregulation has been viewed as a stereotypical step in the formation of regenerative progenitors in the proliferative blastema during salamander limb regeneration (Satoh et al., 2007). *Prrx1* has also been shown to be a marker necessary for maintaining stemness in adult hippocampal tissue (Shimozaki et al., 2013). Recent lineage tracing in the axolotl showed that Prrx1^enh+^-derived cells are multipotent cells that can regenerate skeleton and soft connective tissue (Gerber et al., 2018). The molecular mechanism underlying the enrichment of Prrx1^enh+^ cells within mouse skin wounds is unknown, but live imaging of connective tissue during axolotl regeneration uncovered a pivotal role of cell migration in the process of blastema cell creation (Currie et al., 2016). *Prrx1* has been shown to impact cell migration downstream of focal adhesion kinase (FAK) (McKean et al., 2003) and plays a role in epithelial to mesenchymal transitions (EMT) (Ocaña et al., 2012). In the context of wound healing, the *Prrx1* enhancer could convey an increased readiness or propensity to migrate, amplifying the presence of Prrx1^enh+^ lineage cells in the wound bed.

We specifically took advantage of a previously characterized 2.4 kb enhancer upstream of the Prrx1 transcriptional start site (Durland et al., 2008; Martin and Olson, 2000). Previous transgenics have primarily utilized: (1) a non-inducible *Prrx1-Cre* to label all the descendants of embryonic Prrx1^enh+^ mesenchyme, or (2) an inducible *Cre-ER* to label cells at embryonic timepoints and follow their descendants postnatally. In contrast, we chose to use a *Prrx1-Cre-ER* to convert and label cells specifically at adult stages (7–10 weeks of age) to determine if any adult cell populations retain activity of the embryonic enhancer. Consistent with other work (Moore et al., 2018), we found that the number of cells that retain enhancer activity drops drastically after birth, and by adulthood less than 1% of dermal cells retain sufficient enhancer activity to recombine the LoxP reporter transgene.

We observed a disconnect between the activity of the transgenic *Prrx1* enhancer and the expression of PRRX1 protein in adult dermal cells, such that most dermal cells have easily detectable levels of PRRX1 protein. It will be interesting in the future to understand what molecular factors drive the *Prrx1* enhancer in this small subset of PRRX1^+^ cells during skin homeostasis and injury, and to what degree they overlap with regulators of embryonic *Prrx1* enhancer activity. This could be based on stable extracellular niches that Prrx1^enh+^ cells find themselves in within the perivascular space and the hair follicle dermal papilla (Yeo et al., 2018).

Based on their limited numbers, we wondered if Prrx1^enh+^ cells would function as tissue-resident stem cells to contribute to rejuvenating tissue over time. However, long-term lineage tracing of labeled Prrx1^enh+^ cells over 6 months revealed that these cells remain quiescent within their niches and do not expand to clones larger than 1–3 cells. We next looked at what role Prrx1^enh+^ cells might have during wound healing and injury repair. To our surprise, we observed a strong enrichment of *Prrx1* enhancer lineage cells after full-thickness wounding. This is consistent with injury context of long bone fracture models that tracked Prrx1-Cre lineage cells (Moore et al., 2018; Wilk et al., 2017). The average enrichment of Prrx1^enh+^-derived cells was 16-fold, well above the average density enrichment of PRRX1^+^ dermal cells in the wound bed (1.3-fold). This enrichment of Prrx1^enh+^ lineage cells may have come at the expense of other dermal populations, since Col1a2 lineage cells were depleted from wound sites. We noted a high level of variance between samples, but this could be due to changes in the number of Prrx1^enh+^ cells prior to injury. We sometimes observed concentrated patches of Prrx1^enh+^ cells in ‘uninjured’ skin which may reflect grooming micro-abrasions that occurred between tamoxifen administration and experimental injury.

Because enhancer activity is absent from back skin, which is commonly used for skin injury experiments, we focused on full-thickness skin wounds in the limb. The majority of wound healing assays in murine models are based on back skin wounding due to the inaccessibility during grooming, the ability to make large wounds surpassing 5 mm in diameter and the possibility to splint (to mimic wound healing in humans where there is no contraction). The embryonic source of dorsal cells is diverse and could encompass cells from neural crest, pre-somitic mesoderm and lateral plate mesoderm. In contrast, connective tissue of the limbs derives primarily from a single embryonic source of lateral plate mesoderm. In addition to being of a single embryonic source, experimental paradigms of wound healing in limbs have an important and clinical relevance for diabetic and trauma-related injuries.

Because we only observed a robust amplification in response to tissue injury, we termed these cells and their progeny (which may or may not retain active Prrx1 enhancer) ‘injury responsive’. Recent work has highlighted similar populations of cells that are specifically tuned for tissue repair but not tissue maintenance and growth (Llorens-Bobadilla et al., 2015; Lopez-Baez, 2018; Marecic et al., 2015). Future work will be vital to understand if there is a functional role for Prrx1^enh+^ cells in normal wound repair and if they can have a positive role in regenerative tissue restoration.

One of the most surprising results was the wide contribution of Prrx1^enh+^ lineage cells to subcutaneous tissues under the wound bed. We administered tamoxifen 3 weeks prior to wounding and never observed an enrichment of subcutaneous cells except in the context of wounding and did not observe a similar phenotype when labeling Col1a2^enh+^ fibroblasts. This suggests to us that Prrx1^enh+^ cells from the dermis emigrate into foreign subcutaneous tissues and contribute to adipocytes, fascia and other structures. This resident tissue plasticity may be a conserved feature of Prrx1^enh+^ cells, since axolotl Prrx1^enh+^ cells are able to contribute to new segment formation in contrast to other mesenchymal cell populations (Gerber et al., 2018).

Overall, our results highlight a unique injury-responsive cell population within adult tissue. As impressive as the response of Prrx1^enh+^ cells to injury is, their amplified numbers still make up only a small fraction of the overall population of wound fibroblasts. Future work aimed at understanding the molecular signals that retain and specify Prxx1^enh+^ cells could reveal insights into tipping the balance from scar formation to regenerative tissue restoration.

## MATERIALS AND METHODS

### Transgenic mouse lines

A construct was created containing the 2.4 kb mouse (*Mus musculus*) *Prrx1* enhancer (Logan et al., 2002; Martin and Olson, 2000) followed by the b-globin intron and nuclear-localized teal fluorescent protein-1 (mTFP1-nls), a T2A self-cleaving peptide, and CreERT2. The construct was linearized by digestion by KpnI and injected into the pro-nucleus of Bl6/J mouse oocytes (MPI-CBG Transgenic Core Facility). Six founder animals were generated and F1 progeny were screened for mTFP expression during embryonic limb development (E10.5–E12.5) and the ability to induce recombination of LoxP reporter lines only after administration of tamoxifen. From the initial founder animals, one line was further characterized and used for all subsequent experiments. Further lines used in this study: B6;129S6-Gt(Rosa)26Sor*^tm9(CAG-tdTomato)Hze^*/J (Jackson Laboratory), B6.129S4-Pdgfra*^tm11(EGFP)Sor^*/J (Jackson Laboratory), B6.Cg-Tg(Col1a2-cre/ERT,-ALPP)7Cpd/J (Jackson Laboratory). All mouse lines were bred in Max Planck Institute of Molecular Cell Biology and Genetics (MPI-CBG), Center for Regenerative Therapies Dresden (CRTD) and Research Institute for Molecular Pathology (IMP) facilities. Male and female mice were used for this study. The age of the mice is specified in the individual experiments reported in the results section. All procedures performed in animals adhered to local ethics committee guidelines.

### Immunohistochemistry

Full-thickness skin was dissected from mouse upper forelimb or lower back and attached to filter paper before submersing in 4% PFA in phosphate buffer. Tissue was fixed overnight at 4°C and then washed in PBS and serial overnight washes in 10% and 30% sucrose in PBS before being embedded in OCT and cryo-sectioned in longitudinal sections of 12 µm. For mouse limbs from E18.5–52 weeks of age, limbs were harvested and fixed overnight in PFA, washed in PBS, and incubated with 400 µM EDTA in PBS for approximately 1 week before washing in sucrose, embedding and cryo-sectioning. Standard immunohistochemistry techniques were used for staining. Primary antibodies: PRRX1 (MPI-CBG Antibody facility) used at 1:200, SOX9 (R&D Systems, AF3075) used at 1:200, PERILIPIN (Sigma-Aldrich, P1873) used at 1:200, CD31 (BD Pharmingen, 557355) used at 1:100.

### Wounding

Animals were anesthetized by intraperitoneal injection of a mixture of Ketamine (100 mg/kg) and Xylazin (10 mg/kg). Skin was shaved, chemically defoliated and wiped with 70% ethanol. To create a 2 mm wound in the forelimb, we pulled the skin from the posterior part of the limb (skin in the forelimb is not attached to the muscle-skeletal core) and at the crease, perforated with half of a 2 mm punch biopsy. Wounds were immediately disinfected with iodine solution and wrapped with Tegaderm (3M) and bandage. Animals were given Carprofen at 4 mg/kg and monitored during anesthetic recovery. Wounded animals were monitored daily for signs of infection and any self-removal of bandages. If animals had not removed forelimb bandages and Tegaderm by 48–72 hours post wounding, the dressing was manually removed to synchronize the kinetics of wound resolution. The wound in the posterior area of the limb ensured that the mice were not able to reach it and affect the healing process. We did not observe infection or any complications due to the wound. Animals were euthanized at either 5, 14, 21 or 28 days post wounding and skin was processed as described above.

### Tissue dissociation and FACS

Animals were euthanized and upper arm skin was shaved and cut as full-thickness skin. Adipose depots were manually removed, and the skin was washed once with 70% ethanol and three times with cold sterile PBS. Skin was incubated in 10 mg/ml elastase in DMEM at 37C° for 20 min. Dermal tissue was manually removed and separated from epithelial tissue. Dermal tissue pieces were then placed in 0.35 mg/ml Liberase TM in PBS with 10 units/µl DNase for 37C° for 30 min and then manually dissociated by mechanical disruption with forceps to achieve a single-cell suspension. Cell suspensions were filtered through a 50 µm mesh and diluted in 10% serum containing DMEM. Cell suspensions were centrifuged at 900 relative centrifugal force (RCF) and resuspended in serum-containing DMEM. Both filtered cells and unfiltered tissue pieces were plated on gelatin coated-tissue culture plates. 24 h post-dissociation, both filtered cells and unfiltered tissue were visually assessed for tdTOMATO expression. 24–48 h after dissociation, cells were trypsinized, washed and resuspended in FACs buffer containing 1× PBS (Ca/Mg free), 2 mM EDTA, 25 mM Hepes (pH 7.2), 1% BSA and pen/strep. Non-transgenic controls skin was used to determine gating for tdTOMATO signal.

### Imaging

Images were acquired with a 20× objective Apotome 2 (Zeiss) or inverted laser scanning confocal 780 (Zeiss) at 20 or 40× magnification. Imaging was performed on instruments of the Light Microscopy Facility, a core facility of CRTD at Technische Universität Dresden. Images were analyzed using Fiji and Adobe Photoshop, and alterations to brightness or contrast were applied equally to the entire micrograph for visualization purposes only. Cell quantification was performed in two sections per wound of the mid-area of each animal. A region of interest measuring 1 mm^2^ was cropped in Fiji for quantification. Wound sections were paired with their contralateral control to determine the fold increase and investigator was blinded to the sample grouping.

### Statistical analysis

The means of experimental and control samples were compared by Student's *t*-test (paired). Alpha=0.05 was used to determine statistical significance. Statistical analyses were performed using the Graphpad Prism 7.0 software.

## Supplementary Material

Supplementary information
