# Supplementary material for: The Prrx1 limb enhancer marks an adult subpopulation of injury-responsive dermal fibroblasts
Source: Biol Open. 2019 Jul 5;8(7):bio043711. doi: 10.1242/bio.043711 (PMC6679413; doi:10.1242/bio.043711)
Supplement: Supplementary information [file biolopen-8-043711-s1.pdf]

SUPPLEMENTARY DATA

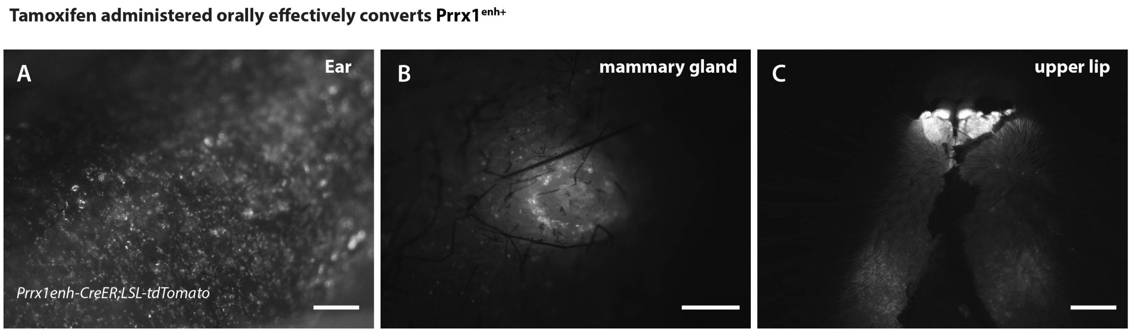

Figure S1.

(A) Tamoxifen administered orally effectively converts cells in ear mesenchyme of *Prrx1enh-CreER;LSL-tdTomato* after a single dose. Scale bar, 500 microns.

(B) Mammary gland. Scale bar, 500 microns.

(C) Intermaxillary portion of the upper lips. Scale Bar, 1 mm.

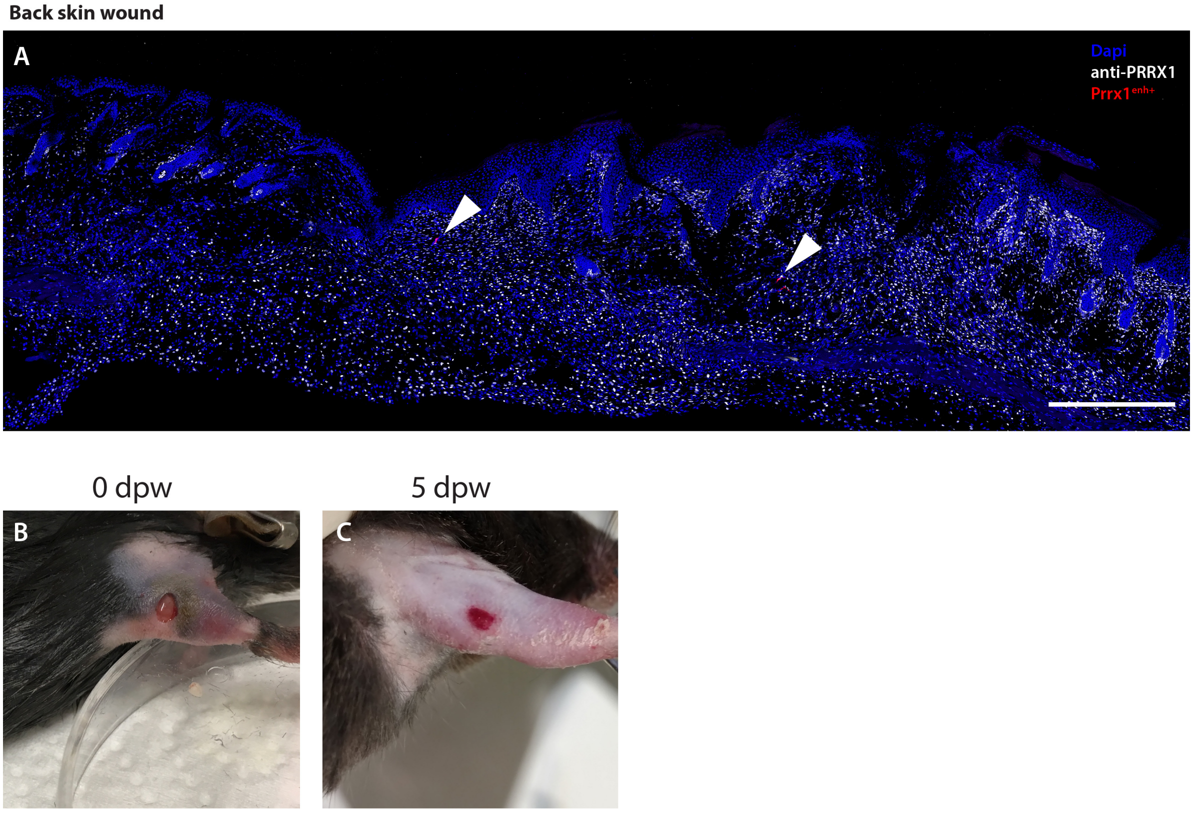

Figure S2.

(A) 2 mm full-thickness wounds in the dorsal trunk of *Prrx1enh-CreER;LSL-tdTomato* mice produce 0-10 labeled cells in a cubic centimeter of wound tissue. Scale bar, 500 microns.

(B) A fresh 2 mm full-thickness wound in the posterior skin of the upper limb. The position of the wound does not enable splinting of the wound, therefore, we used semi-occlusive dressing to protect the wound from infection and delay the contraction of the wound.

(C) After 5 days post wounding the semi-occlusive dressing is removed as healing proceeds.

Biology Open • Supplementary information

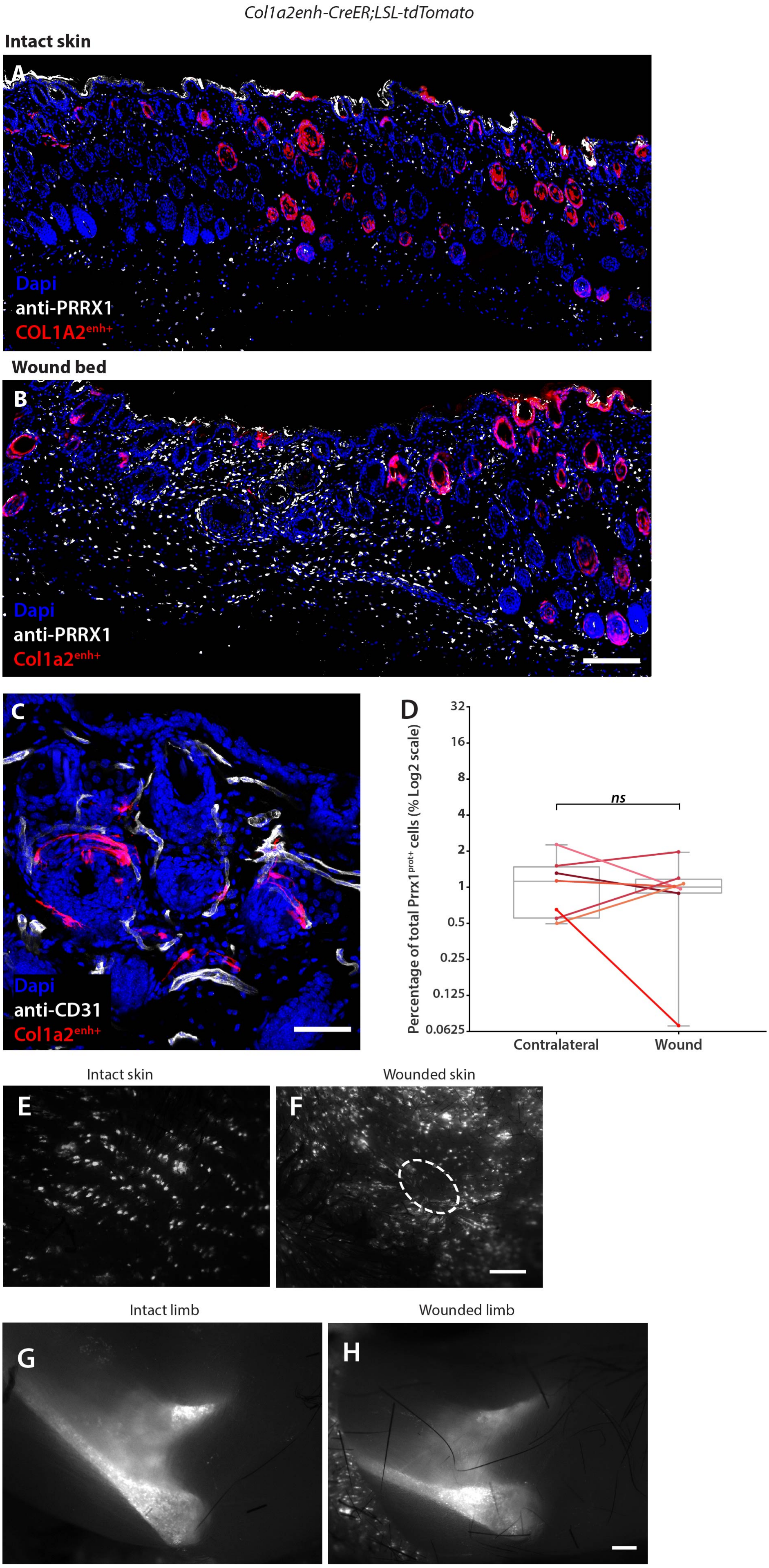

Biology Open • Supplementary information

Figure S3.

(A) Intact skin of *Col1a2enh-CreER;LSL-tdTomato* mice 3 weeks after administration of tamoxifen. The majority of the labelled cells are in epidermal layers with some dermal cells visible. Scale bar, 200 microns.

(B) A 2 mm full thickness wounds in the posterior skin of the upper limb of *Col1a2enh-CreER;LSL-tdTomato.* $Col1a2^{enh+}$ cells are mostly absent in the wound bed. Scale bar, 200 microns.

(C) $Col1a2^{enh+}$ cells are not associated to blood vessels. Scale bar, 50 microns.

(D) Quantification of $Col1a2^{enh+}$ cells in sections from paired samples of contralateral and wounded limb skin. The percentage of $Col1a2^{enh+}$ from the total $PRRX1^{+}$ of cells/mm^2 (box is mean and 95% CI, error bars min and max values) are plotted. Y axis is shown in log2 for optimal visualization of values below 1. Y-axis scale is standardized to that of $Prrx1^{enh+}$ cells counted in Figure 3C.

(E) Stereoscope micrograph of skin from an intact limb.

(F) Skin from wounded limb 21 days after wounding. $Col1a2^{enh+}$ cells are mostly absent in 2 mm full thickness wounds in the limb, highlighted by dashed oval. Scale bar, 1 mm.

(G) Fixed limb of mouse after skin collection in contralateral limb.

(H) Fixed limb of mouse after skin collection in wounded limb. Positively labeled cells in the bone and skin and absent in superficial subcutaneous tissue under the wounded dermis. Scale bar, 500 microns.

Biology Open • Supplementary information
